# Dynamic Advances in Emotion Processing: Differential Attention towards the Critical Features of Dynamic Emotional Expressions in 7-Month-Old Infants

**DOI:** 10.3390/brainsci10090585

**Published:** 2020-08-24

**Authors:** Shira C. Segal, Margaret C. Moulson

**Affiliations:** Department of Psychology, Ryerson University, Toronto, ON M5B 2K3, Canada; mmoulson@ryerson.ca

**Keywords:** infant eye tracking, emotion processing, visual scanning, visual attention, emotional development

## Abstract

Infants’ visual processing of emotion undergoes significant development across the first year of life, yet our knowledge regarding the mechanisms underlying these advances is limited. Additionally, infant emotion processing is commonly examined using static faces, which do not accurately depict real-world emotional displays. The goal of this study was to characterize 7-month-olds’ visual scanning strategies when passively viewing dynamic emotional expressions to examine whether infants modify their scanning patterns depending on the emotion. Eye-tracking measures revealed differential attention towards the critical features (eyes, mouth) of expressions. The eyes captured the greatest attention for angry and neutral faces, and the mouth captured the greatest attention for happy faces. A time-course analysis further elucidated at what point during the trial differential scanning patterns emerged. The current results suggest that 7-month-olds are sensitive to the critical features of emotional expressions and scan them differently depending on the emotion. The scanning patterns presented in this study may serve as a link to understanding how infants begin to differentiate between expressions in the context of emotion recognition.

## 1. Introduction

The ability to process and recognize emotions is critical in guiding successful interactions with other individuals [1]. Given the importance of this skill, it is not surprising that its development begins within the first few days after birth and continues throughout infancy and beyond. Learning to orient attention to faces and to process facial expressions are all part of the skill set required by infants for communicating with caregivers and navigating their social environment [2]. Awareness of others’ emotions is also crucial for the development of intersubjectivity (i.e., shared mental experience between two or more individuals), which is a key component of socio-emotional development [3]. Emotion recognition is therefore a foundational skill that supports decoding social cues and understanding complex social interactions [4]. 

Previous research has outlined a general timeline for how emotion recognition progresses across the first year. Three-month-old infants, and potentially even newborns, are able to discriminate among a limited number of emotional expressions and display a preference for happy expressions [5]. By 5 to 7 months, infants begin to exhibit more sophisticated abilities with respect to decoding the underlying affective information being portrayed in various emotional expressions. For example, infants of this age demonstrate the ability to identify multiple representations of the same emotion as part of a cohesive category [6] and are able to detect a common emotion across visual and auditory stimuli (i.e., faces and voices; [7,8,9]). Between 9 and 12 months of age, infants exhibit progressively more advanced emotion recognition. Specifically, social referencing—the ability to modify and regulate behaviour according to others’ facial expression—emerges around this time [10]. 

This timeline provides us with a general framework for how emotion recognition progresses across the first year, but a number of questions remain unanswered. In particular, we know little about the underlying mechanisms that facilitate infants’ advances in emotion recognition in the first year of life. For example, could infants’ visual attention patterns towards emotional expressions reveal clues about concurrent advances in emotion recognition ability as they reach the age in which they improve in this ability? 

Eye tracking is a method that can provide insight into this potential mechanism—the visual scanning strategies employed by infants in response to faces in their environment. Previous studies have found relations between visual scanning strategies and outcome measures of face and emotion discrimination. For example, Amso, Fitzgerald, Davidow, Gilhooly, and Tottenham [11] found that 6-to 11-month-olds’ greater allocation of attention to the eyes of a fearful face during habituation was correlated with a novelty preference for happy faces on subsequent trials—despite the overall sample failing to exhibit a novelty preference. Thus, without examining scanning patterns, the results would suggest that infants at this age were unable to detect the differences between a fearful and happy face. However, with the addition of examining scanning patterns, it is revealed that the infants who focused their attention towards the fearful eyes were able to visually discriminate between these expressions. Thus, these results highlight the utility of examining scanning patterns as a method of tracking the information that infants use to learn about emotional expressions in real time, as well as how this information relates to outcomes such as visual discrimination. Similarly, Xiao et al. [12] examined the role of visual scanning patterns in relation to face recognition in a sample of 3-, 6-, and 9-month-old infants. They found that infants who exhibited greater fixation shifting during the encoding phase had better recognition for those faces during the test trial, but only for the older infants (6- and 9-month-olds). Thus, individual differences in infants’ scanning patterns seem to be related to individual differences in discrimination of faces and facial expressions, suggesting that examining how infants deploy their visual attention offers an important window into their developing face and emotion recognition skills. 

Numerous studies have previously used eye tracking to delineate the ways in which adults, and to a lesser degree infants, scan emotional expressions. In adults, the eye region is heavily attended to in the decoding of facial expressions [13,14]; however, facial features tend to vary in their relative importance to recognition depending on the emotion. The eye region is relied upon for adults’ recognition of fearful, neutral, angry, and sad faces, while the mouth region is considered to be critical in the recognition of happy faces [13,15,16,17,18,19,20]. 

The eyes also capture attention in infants. Within hours after birth, newborns prefer looking at pictures of people with open eyes compared to closed eyes [21], and by 2 months of age, infants devote increased attention to the eyes when scanning faces [22]. Although younger infants continue to allocate disproportionate attention towards the eye region, they begin to exhibit more distributed scanning across all facial regions (e.g., the mouth) towards the end of the first year of life [2,23].

Studies examining infants’ scanning patterns of *emotional* faces are more limited and provide somewhat inconsistent findings. For example, Miguel, McCormick, Westerlund, and Nelson [24] found that 5-, 7-, and 12-month-olds exhibited greater looking toward the top of the face (i.e., the eyes and forehead) when scanning fearful and angry compared to happy faces, and greater looking to the bottom of the face (i.e., mouth and chin) when scanning happy compared to fearful and angry faces. Additionally, Gredebäck, Eriksson, Schmitow, Laeng, and Stenberg [25] found that 14-month-old infants displayed a greater number of fixations and distributed their gaze more broadly when scanning fearful compared to happy and neutral expressions. These studies thus provide support for differential scanning patterns depending on the emotional expression. In contrast, Peltola, Leppänen, Vogel-Farley, Hietanen, and Nelson [26] did not report differential scanning patterns across expressions (fearful, happy, and neutral). Instead, they reported that 7-month-olds spent 53% of their time scanning the eye region, 16% scanning the nose, and 4% scanning the mouth across *all* expressions. Similarly, Hunnius, de Wit, Vrins, and von Hofsten [27] also found that 7-month-olds exhibited comparable scanning of the eye region across angry, fearful, happy, sad, and neutral expressions. The importance of the eye region is further highlighted by findings that 7-month-olds exhibited the greatest proportion of scanning the eye region compared to the mouth region when happy and fearful expressions were presented subliminally (50 ms) compared to supraliminally (900 ms), thus suggesting that this feature may be relied upon for the quick extraction of affective information [28]. 

Although these studies provide a general framework for understanding how infants scan *static* faces, there has been a recent push for the inclusion of more ecologically valid facial stimuli in studies of infant emotion recognition [29,30]. Facial expressions are dynamic in nature; thus, the use of dynamic stimuli may produce more reliable results, as they mimic infants’ experience with emotions in the real world [31,32]. Thus, a major limitation of past research on infants’ processing of emotional expressions is that the majority of studies to date have used static images of faces. The reliance on static images may have led researchers to underestimate infants’ emotional processing abilities, as these images may relay less information than infants typically have access to when navigating emotions in their day-to-day experiences. Caron, Caron, and Myers [33] noted that emotional expressions are behavioural events that unfold over time, and as such, important diagnostic information is likely transmitted in the patterns of change and movement in facial musculature, body, and voice (i.e., diagnostic for accurate emotion judgments). Indeed, infants can recognize facial expressions from point-light displays of facial movement alone [34], thus highlighting that facial movement is an integral component of emotional expressions [35]. 

Given these insights, exploring how infants scan *dynamic* emotional faces is a critical methodological shift that is required to better understand how infants visually process emotional expressions. Very few studies have investigated infant visual scanning of dynamic emotional faces, yet we know that infants scan dynamic faces differently than static faces. Specifically, infants have been found to exhibit longer looking times to dynamic compared to static faces [36], exhibit increased scanning of internal versus external facial features when faces are presented dynamically [29], and learn unfamiliar faces at a quicker rate when presented with dynamic compared to static stimuli [37]. These studies are important because they demonstrate that motion impacts the way that infants scan faces, which can have broad implications for their subsequent processing and learning of facial information. What is less clear are the implications of using dynamic stimuli in studying infant emotion recognition. The current literature is lacking a basic delineation of infants’ scanning patterns when presented with dynamic emotional expressions. 

In one such study, Soussignan et al. [38] used eye tracking to examine developmental changes in 3-, 7-, and 12- month-old infants’ scanning of basic emotions (anger, disgust, fear, sadness, happiness, and neutral) using short movie clips of 3D avatar faces. They found that 7-month-olds exhibited greater attention towards the eye region of angry compared to happy faces, as well fearful compared to neutral faces, and 12-month-olds exhibited greater scanning of the fearful eyes compared to the eyes of all other expressions. Regarding the mouth region, they found that 7- and 12-month-olds fixated longer to the mouth of happy faces compared to all other expressions. Comparatively, 3-month-olds attended to facial features in a similar manner regardless of emotional expression and spent more time scanning the external facial features compared to the 7- and 12-month-olds. Although these findings provide evidence of differential scanning of facial expressions across the first year, the use of virtual models precludes our understanding of infants’ visual scanning patterns for human faces. To our knowledge, no other studies have investigated infants’ visual scanning of dynamic emotional faces.

The current study will examine 7-month-old infants’ exploratory scanning patterns when presented with dynamic expressions of anger, fear, happiness, neutral, and sadness. We chose to test 7-month-old infants, because 7 months seems to be a key age in the development of emotion recognition, with robust abilities in place for recognizing emotional expressions across different identities, emotional intensities, and multiple modalities [8,39,40]. Although this study does not examine any specific outcome measures of emotion recognition, achieving a more fine-grained understanding of infants’ visual exploratory patterns of dynamic emotional expressions is a critical building block towards understanding how advances in emotion recognition develop at this age. As such, this study will focus on quantitatively describing scanning patterns employed by infants when presented with dynamic emotional expressions. We characterize infants’ scanning patterns using multiple measures, including the percentage of looking time towards critical features that have been implicated in emotion recognition in adults (i.e., eyes and mouth) and a time-course analysis of looking to the critical features across consecutive 200 ms time windows to understand how infants’ attention evolves over time.

We hypothesized that infants would exhibit differential scanning patterns for angry, fearful, happy, neutral, and sad faces. Specifically, we predicted that infants would fixate longer to the eye region than the mouth region of angry, fearful, neutral, and sad faces, and longer to the mouth region than the eye region of happy faces. The planned analysis regarding the time-course analysis was exploratory in nature and was intended to aid in further characterizing differences in infants’ scanning patterns in response to different emotional expressions. As such, we did not have specific predictions regarding this analysis.

## 2. Materials and Methods

### 2.1. Participants

A total of 63 infants participated in this study (M age = 219 days, SD = 27.54, 33 females). Infants were all born full term (37–42 weeks gestation), and parents reported no visual impairment. Parents reported infants’ race as White (56%), Asian (13%), Black (3%), Latin American (3%), and multiracial (25%). Information on the socioeconomic status of the participants was not available. Parents of infants were contacted from an internal database via phone and email regarding participation in the current study. The sample size was determined by a power analysis using G*Power 3 software (Version 3.1.9.3, Heinrich Heine University, Düsseldorf, Germany) [41], to ensure sufficient power to detect a medium effect in the within-between analysis of variance (ANOVA) interaction (given the statistical significance criterion of 0.05, based on previous data; [38]). An additional 11 participants were tested, but their data were excluded because they did not complete the task due to fussiness (leading either to poor calibration at the start of the task, or inattention during the task; *n* = 5), technical difficulties with the eye tracking equipment (*n* = 5), or they looked at the face for less than 1000 ms for both trials showing the same emotion (*n* = 1). Parents or legal guardians provided written consent for their infants to participate in the study. 

### 2.2. Stimuli and Apparatus

The stimuli consisted of validated dynamic videos of five White female models expressing angry, fearful (teeth visible), happy (open mouth; teeth visible), neutral, and sad emotional expressions from the Amsterdam Dynamic Facial Expression Set (ADFES; [42]). According to a validation study, the raw accuracy (%) and unbiased hit rates (*H_u_*) for each emotion collapsed across models are the following: anger (92%, *H_u_* = 0.87), happiness (95%, *H_u_ =* 0.81), fear (87%, *H_u_ =* 0.80), and sadness (90%, *H_u_ =* 0.79; [42]). In each video, the model begins with a neutral expression, shifts to the peak intensity between 500 and 1000 ms, and remains at this peak intensity for another 5000 ms. Thus, each video lasted for 6000 ms in total. All models were facing forward in these videos. Infants were presented with two different models displaying each of the five emotional expressions for a total of 10 trials. As such, infants viewed all 5 expressions presented by the first model, followed by each expression presented by the second model. Expressions were presented in a randomized order within each model identity, and exposure to each model was counterbalanced across participants.

The remote Eyelink 1000 Plus (Arm Mount, SR Research Ltd., Ottawa, ON, Canada) was used to record eye-tracking data. The equipment included an adjustable arm mount, a 22″ LCD monitor, and the camera (16 mm lens) and illuminator held beneath it. Data were collected using the remote monocular mode, which allows for gaze position to be recorded at a sampling rate of 500 Hz without head stabilization. This is the optimal setting when the chin rest or head mount are not available, such as for use with infants. The gaze tracking range is 32° horizontally, and 25° vertically, with allowed head movements of 40 × 40cm at 70cm (horizontal × vertical × depth) without reductions in accuracy. The reported spatial resolution (precision) for the tracker is 0.01° when used in remote mode and when participants are seated 40–70 cm from the screen. The Eyelink system defines fixations and saccades using a velocity-based parsing algorithm. Instantaneous velocity and acceleration data are recorded for each data sample picked up by the eye tracker, and these values are compared to established thresholds (velocity: 40 degrees/s; acceleration: 8000 degrees/s^2^). Data samples whose values fall above these thresholds are classified as saccades, and samples whose values are below these thresholds are classified as fixations. The primary variable that we relied on to calculate our looking times (dwell time) only includes fixations and automatically filters out saccades.

### 2.3. Procedure

All procedures were approved by the Research Ethics Board of the university and the study was conducted in accordance with the Declaration of Helsinki. Infants were seated on their parent’s lap, and the researcher ensured that they were seated at an approximate distance of 60 cm from the screen. The researcher placed a sticker on the infant’s forehead to serve as a target for the eye tracker to establish focus. The researcher first proceeded with calibration (3-point model with animated targets), to establish the corneal reflection (CR) detection threshold. Following successful calibration, the researcher started the trial. Infants were presented with one video on the screen at a time, which played for 6 s. Each face subtended a visual angle of 10.48° horizontally, and 12.84° vertically. Each infant was presented with 10 trials in total, with an animated attention-grabber appearing in the center of the screen after each trial to re-focus infants’ attention. There was a 2-s gaze contingency built into the attention-grabber to ensure that a new trial only began once infants fixated the center of the screen, and that the eye-tracking signal was strong enough to be detected between trials. If the signal was not detected during the presentation of the attention-grabber, the researcher re-calibrated before the next trial could proceed.

### 2.4. Data Reduction

#### 2.4.1. Creation of Interest Areas and Report Generation 

**Figure 1:**
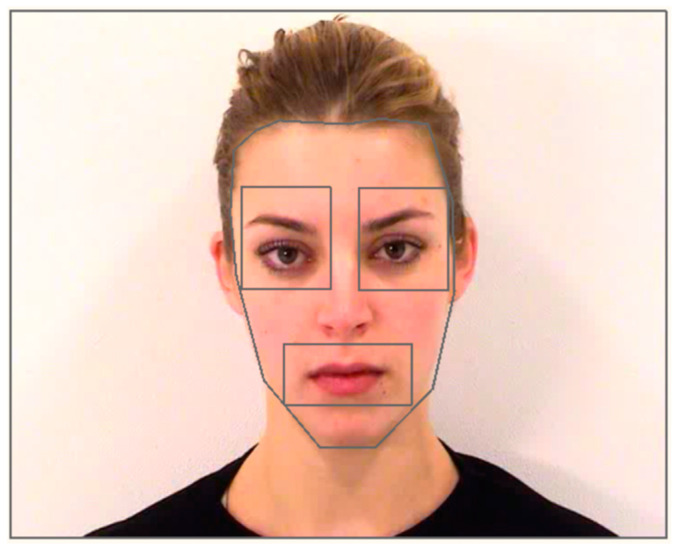
Interest areas (IAs) for model 1.

Interest areas (IAs) were created in Eyelink Data Viewer version 3.1 (SR Research Ltd., Ottawa, ON, Canada). IAs were manually created using a combination of rectangles and free hand ellipses. The 5 IAs created were: left eye, right eye, mouth, face, and screen (Figure 1).

IAs were created so that the area of space for each feature was the same across all expressions for the same model (e.g., area for the mouth region was consistent across all expressions for model 5 but differed from the IAs of model 3). Static IAs were used, given that the head remained in the same position for the duration of the trial, so it was possible to cover the facial features in their entirety and capture subtle movements in each feature (e.g., eyes blinking, mouth movement) within the same IA throughout the trial. A listing of the area of all IAs is presented in Table A1 and Table A2 in Appendix A. 

After IAs were created, they were uploaded to the respective session trials, and Data Viewer was used to generate IA reports. These reports specified the raw dwell time of fixations (ms) for each IA within the trial period. Considering that raw dwell time is a less meaningful metric than a percentage of looking time compared to the total time spent scanning the face, a MATLAB script (MathWorks, Natick, MA, USA) was employed to divide the dwell time of each feature IA by the face IA (i.e., out of the total dwell time to the face, what percentage of that time was spent fixating various features?). 

#### 2.4.2. Sufficient Looking to the Face 

To examine whether trials should be excluded on the basis of insufficient looking towards the face, the raw dwell time to the face IA was examined across each trial for each participant. Any trials where the infant spent less than 1000 ms scanning the face region were excluded from analysis. This resulted in the exclusion of 16 trials across 12 participants. In cases where trials were removed, the single trial of a particular emotion was used instead of the average. All infants had at least one trial for each emotion. 

## 3. Results

The average calibration accuracy across the sample was 0.72° error in the visual angle (SD = 0.26°; precise calibration values were unavailable for *n* = 3 infants; however, the task was never initiated if an infant had poor calibration). 

### 3.1. Looking to the Face across Trials and Emotions

The subsequent statistical analyses were conducted using IBM’s Statistical Package for the Social Sciences (SPSS) version 24 (IBM Corporation, Armonk, NY, USA). The average looking time to the face collapsed across all trials and emotions was 4515.78 ms (SD = 1278.39). A one-way ANOVA was conducted to examine whether attention waned across the 10 trials. Mauchly’s test indicated that the assumption of sphericity had been violated, so the Greenhouse–Geisser correction was applied. The effect of trial was not significant, F(6.95, 430.80) = 1.89, *p* = 0.070, η^2^ = 0.03, indicating that there was comparable looking time across trials. As such, stimulus order was not included as a factor in the following analyses. 

Trials were then sorted by emotion, and another one-way ANOVA was conducted to examine whether there were differences in looking towards the face by emotion. Looking towards the face ranged between 4329.73 and 4737.39 ms across expressions. Mauchly’s test indicated that the assumption of sphericity had been violated, so the Greenhouse–Geisser correction was applied. The effect of emotion was significant, F(3.38, 209.77) = 3.02, *p* = 0.025, η^2^ = 0.05. For the simple main effects follow-up, the Benjamini–Hochberg correction for multiple comparisons was applied [43] using a False Discovery Rate (FDR) of 0.05. This analysis revealed that infants exhibited greater scanning of fearful faces (M = 4737.39, SD = 963.86) compared to neutral (M = 4329.73, SD = 982.50; *p* = 0.006; adjusted *p* = 0.04) and angry faces (M = 4430.74, SD = 1021.49, *p* = 0.008; adjusted *p* = 0.04). 

### 3.2. Percentage of Dwell Time towards Critical Features

To examine whether infants differentially attended to the critical features (i.e., eyes and mouth) depending on the emotional expressions, A 2 × 5 repeated measures ANOVA was conducted on the percentage of dwell time towards the critical facial features (eyes vs. mouth) for each emotion (anger vs. fear vs. happiness vs. neutral vs. sadness). The percentage of dwell time towards the critical features was calculated as the amount of time infants spent fixating to the critical feature divided by infants’ overall dwell time towards the face. This percentage was first calculated for each trial before it was averaged across the two trials of a single emotion to provide a single percentage of dwell time for the feature. The right and left eye IAs were combined to create a single measure of looking to the eye; however, because the size of the combined eye region was larger than the mouth region, the percentage of dwell time towards the eyes was divided in half. This size-corrected variable for dwell time towards the eye was used for subsequent analyses. 

Mauchly’s test indicated that the assumption of sphericity had been violated, so the Greenhouse–Geisser correction was applied. The main effect of emotion was significant, F(2.85, 176.56) = 9.18, *p* < 0.01, η^2^ = 0.13. Simple main effects were conducted with the Benjamini–Hochberg correction for multiple comparisons using a False Discovery Rate (FDR) of 0.05. Across both features, infants exhibited a greater percentage of dwell time towards happy faces (M = 15.82, SD = 10.14) compared to all other emotions (anger: M = 10.73, SD = 5.84; fear: M = 12.19, SD = 6.29; neutral: M = 10.91, SD = 4.64; and sadness: M = 11.47, SD = 6.53, *p*s <.002; adjusted *ps* <.01). The main effect of feature was not significant, F(1, 62) = 2.03, *p* = 0.159, η^2^ = 0.032. Across all five emotions, infants exhibited a similar percentage of dwell time to the eyes (M = 13.74 SD = 5.72) and the mouth (M = 10.71, SD = 12.53).

**Figure 2:**
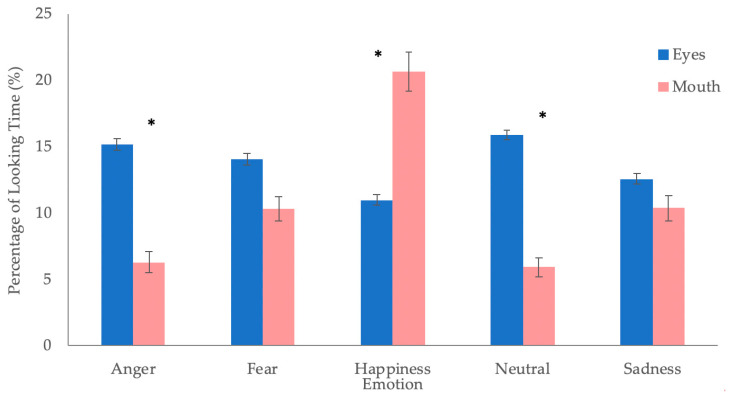
Percentage of looking time towards the critical features (eyes, mouth) for each of the five emotions. (*n* = 63) * *p* < 0.01, adjusted *p* < 0.02.

The expected interaction between emotion and feature was significant, F(2.76, 171.37) = 23.47, *p* < 0.001, η^2^ = 0.28 (Figure 2). Simple main effects were conducted with the Benjamini–Hochberg correction for multiple comparisons (FDR = 0.05). Post-hoc analyses revealed that infants exhibited a greater percentage of dwell time to the eyes than the mouth for angry (*p* < 0.001; adjusted *p* < 0.001) and neutral (*p* < 0.001; adjusted *p* < 0.001) faces and a greater percentage of dwell time to the mouth than the eyes for happy faces (*p* = 0.008; adjusted *p* = 0.02). There were no significant differences in looking to the eyes or mouth for fearful (*p* = 0.131; adjusted *p* = 0.218) and sad faces (*p* = 0.372; adjusted *p* = 0.465).

### 3.3. Time-Course Analysis

To supplement the average dwell time analysis, which examines infants’ *overall* patterns of looking to the eyes and mouth over the entire trial, a time-course analysis was conducted to more thoroughly examine how infants’ attention towards the eye and mouth region evolved over the course of the trial. Rather than examining dwell time averaged across the entire trial, this analysis allows for a moment-by-moment examination of the ways in which infants’ scanning patterns evolved as the facial expression emerged and once the peak expression was reached. For this analysis, the trial was segmented into 200 ms intervals to examine the likelihood of infants looking to the eyes and mouth within each consecutive time window of the trial. This time window was specifically chosen to represent the approximate time that it takes infants to initiate a saccade [44]. The parameters were further set to exclude eye-tracking samples during saccades and time windows that contained non-fixation samples, such that the final output detailed the percentage of fixations to either the eyes or mouth region per each 200 ms of the trial. For the following analyses, we decided to only examine the first half of the trial (0–3000 ms; first 15 time bins), as we wanted to capture infants’ looking patterns during the portion of the trial when the faces shifted from neutral to expressive as well as a few seconds after, when the expressions were held constant. The number of infants who contributed at least one eye and one mouth fixation in each 200ms-time-bin are presented in Appendix A (Table A3 and Table A4).

**Figure 3:**
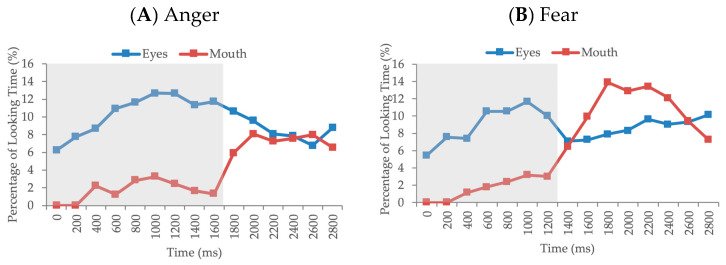

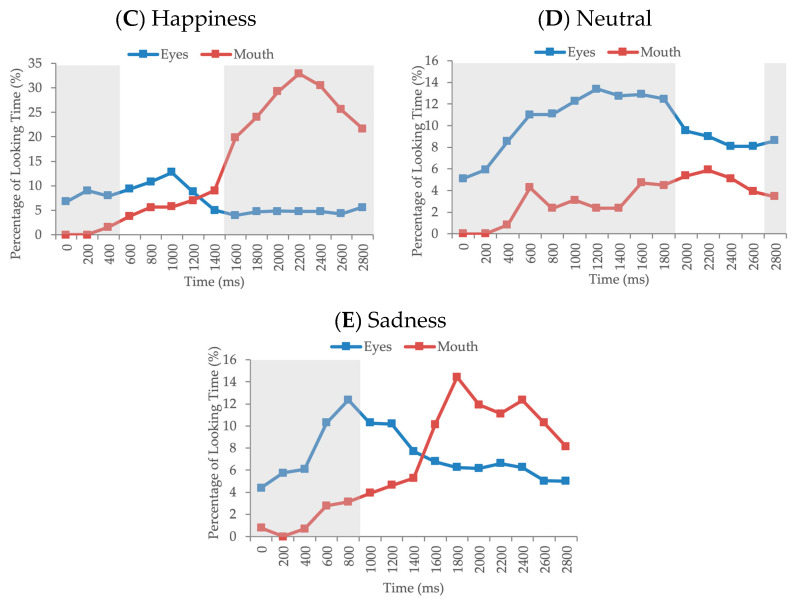
Time-course analysis: percentage of looking time to the eyes and the mouth over the first 3000 ms of the trial presented for (**A**) Angry; (**B**) Fearful; (**C**) Happy; (**D**) Neutral; and (**E**) Sad faces (*n* = 63). Time intervals with significant differences are highlighted in the grey boxes (*p*s < 0.016; adjusted *p*s < 0.049).

In order to statistically compare the evolution of looking to the eyes and mouth across the trial for each emotion, a series of paired-sample *t*-tests were conducted between the eyes and mouth within each consecutive time window. The Benjamini–Hochberg procedure was used to control for multiple comparisons (FDR = 0.05) across these analyses. For angry faces, infant exhibited significantly greater looking to the eyes compared to the mouth between 0 and 1800 ms (*t*s > 3.03, *p*s < 0.004; adjusted *p*s < 0.02). For fearful faces, there was significantly greater looking to the eyes compared to the mouth between 0 and 1400 ms (*t*s *>* 3.20, *p*s < 0.002; adjusted *p*s < 0.015). For happy faces, looking to the eyes was significantly greater between 0 and 600 ms (*t*s *>* 3.08, *p*s < 0.003; adjusted *p*s < 0.015), and looking to the mouth was greater between 1600 and 3000 ms (*t*s *>* −0.349, *p*s < 0.001; adjusted *p*s < 0.008). For neutral faces, infants exhibited greater looking to the eyes compared to the mouth between 0 and 2000 ms (*t*s *>* 2.57, *p*s < 0.013; adjusted *p*s < 0.049) and between 2800 and 3000 ms (*t =*2.48, *p* = 0.016; adjusted *p* = 0.048). Finally, for sad faces, infants exhibited greater looking to the eye region compared to the mouth region between 0 and 1000 ms (*t*s *>* 2.86, *p*s < 0.006; adjusted *p*s < 0.03; Figure 3). Degrees of freedom were 62 across all paired-samples *t-*tests. 

### 3.4. Visual Salience Analysis

**Figure 4:**
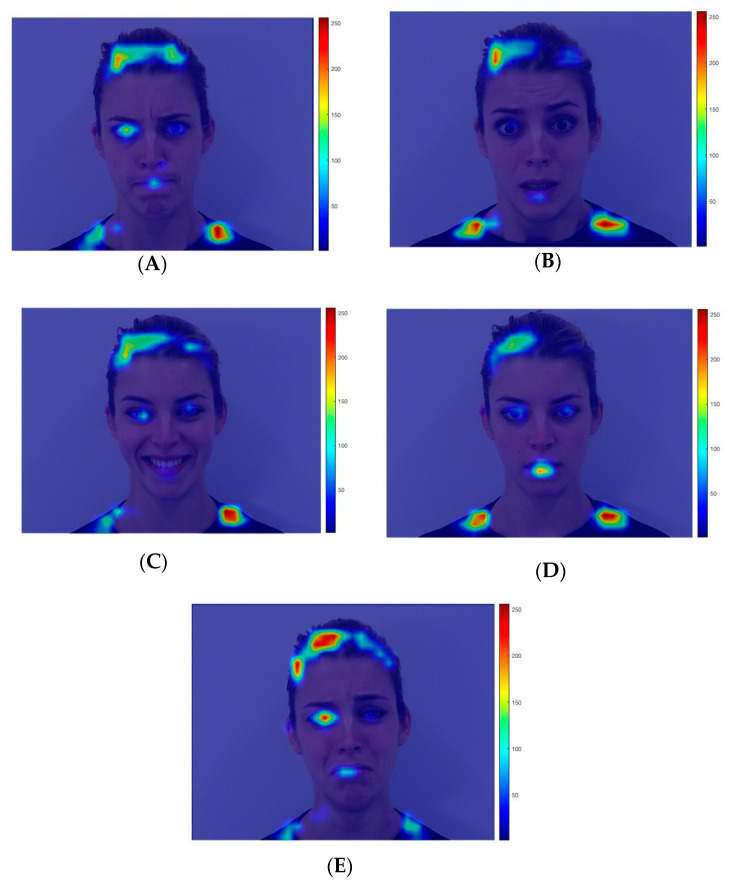
Heat maps representing visual saliency derived from the Matlab Saliency Toolbox [45] overlaid on (**A**) Angry; (**B**) Fearful; (**C**) Happy; (**D**) Neutral; and (**E**) Sad peak facial expressions of one identity. Hot colors (e.g., red, yellow) represent higher saliency locations and cool colors (e.g., blue) represent lower saliency locations.

Recognizing that a compelling interpretation of the current findings is that infants’ looking patterns may be driven by low-level visual properties of the stimuli, we conducted a visual salience analysis of each stimulus using the Matlab Saliency Toolbox [45]. By assessing the low-level visual properties of an image (e.g., color, intensity), this toolbox produces a “heat map” that highlights visually salient areas of an image. For each of the 25 dynamic stimuli used in the current study (5 identities × 5 facial expressions), we used an image of its peak expressiveness [42]. Each of these images was analyzed using the Saliency Toolbox. Saliency maps for one identity are portrayed in Figure 4 and the saliency maps for all of the identities can be found in Supplementary Figure S1. The saliency maps are relatively consistent across different facial expressions, with external features (e.g., hairline, shoulders) and the eyes (particularly the left eye from the viewer’s perspective) appearing as the most visually salient features. Notably, the mouth region of the happy facial expression was not noticeably more salient than the mouth region of the other facial expressions; in fact, the mouth region appeared *more* salient in the fearful, sad, and/or neutral expressions compared to the happy facial expression for several of the identities. This finding suggests that infants’ enhanced attention to the mouth of the happy facial expressions (compared to the other facial expressions) may not be explained by the low-level visual salience of the mouth region alone.

However, simply viewing the saliency maps and making a subjective judgment may obscure subtle differences in the low-level visual salience of various features in the different emotional expressions. Therefore, in order to quantify the visual salience of the eyes and mouth in the different facial expressions, we used the “winner-take-all” algorithm from the Saliency Toolbox, which identifies visually salient areas of the image in order from most to least salient. For each image, we identified the ranking of the left eye, right eye, and mouth in terms of the most salient area of the image (e.g., for Face A, the left eye was identified as the fourth-most salient area). Table 1 displays the median saliency value across identities for each interest area (left eye, right eye, mouth) in each facial expression.

## 4. Discussion

The goal of the current study was to investigate 7-month-old infants’ visual scanning of dynamic emotional expressions. Infants were presented with sequential displays of angry, fearful, happy, neutral, and sad dynamic faces while an eye tracker recorded the duration of gaze towards various facial features. The main hypothesis was supported, such that infants exhibited differential scanning of emotional expressions. In line with our predictions, infants exhibited more fixations to the eyes than the mouth of angry and neutral expressions, and they exhibited greater looking to the mouth than the eyes of happy faces. In contrast to our predictions, infants exhibited similar looking to the eyes and mouth of fearful and sad faces overall. The time-course analysis provided further insight into *when* during the trial differences in looking to the eyes and mouth emerged within each emotion. For all emotions, infants allocated significantly greater attention to the eyes compared to the mouth at the start of the trial, but the duration of greater looking to the eyes differed across emotions (e.g., ranging from 600 ms up to 2000 ms). For happy faces, a sharp increase in looking to the mouth emerged around 1600 ms, after which time there was significantly greater looking to the mouth region. The happy expression was the only expression for which there was increased looking to the mouth compared to the eyes at any point in the trial.

Although the extant literature on processing of dynamic facial expressions of emotion is limited, these findings are generally in line with previous data suggesting that by 7 months of age, infants exhibit scanning patterns that take into account the critical features of dynamic emotional expressions. Soussignan et al. [38] found that while 3-month-old infants exhibited similar scanning patterns across multiple expressions, 7-month-olds exhibited differential scanning of facial features across expressions (e.g., increased fixations towards the eyes of fearful, angry, and sad faces compared to happy faces, fearful compared to neutral faces, and increased fixations towards the mouth of happy faces compared to all other expressions). Similarly, adults typically allocate attention towards different facial features depending on the emotion. Previous data suggest that the eyes are a critical feature in the recognition of fearful, neutral, angry, and sad faces, while the mouth region is more critical in the recognition of happiness [13,15,16,17,18,20]. While these studies have been informative in identifying critical features for recognition, most have relied on static facial images and have been conducted with adults. Therefore, the current study is an important extension of this literature. Our finding of differential allocation of attention towards these same features of dynamic angry, neutral, and happy expressions suggests that by 7 months of age, infants are sensitive to the facial features that are diagnostic of a given facial expression and scan dynamic expressions in a manner consistent with how adults scan static expressions. 

Although we did not find evidence of overall greater scanning of the eyes compared to the mouth region for fearful and sad faces, as hypothesized based on previous literature with static expressions, the results of the time course analysis suggest that the eye region is still an important region of focus in the scanning of these expressions, as infants exhibited greater attention to the eyes at the beginning of the trial for both expressions. This difference highlights the importance of investigating infant scanning patterns with dynamic, ecologically valid expressions, where facial motion may alter infants’ scanning of expressions compared to our current knowledge based on static images. The addition of our visual salience analysis provides further confidence that infants’ scanning patterns were not simply driven by one important aspect of salience, the low-level visual properties of the face stimuli (e.g., passively attending to the high contrast areas of the face) and, rather, may represent a more active pattern of scanning that is driven by a sensitivity to diagnostic facial features.

Previous studies using dynamic facial expressions have reported mixed evidence regarding whether facial movement facilitates infants’ recognition ability. For example, Ichikawa et al. [46] found that 6- to 7-month-old infants were only able to recognize a subtle happy expression when it was presented dynamically, rather than statically. In contrast, when Ichikawa and Yamaguchi [47] measured infants’ recognition of a subtle anger expression, they found that infants could not recognize this expression when presented dynamically, even though they could recognize it in static form. Thus, it is possible that facial movement sometimes enhances infants’ recognition of emotion and other times disrupts encoding of emotional information, especially for emotions with which infants typically have less experience (e.g., anger). Although the current findings do not speak directly to this possibility, they suggest that infants display scanning patterns that are consistent with encoding the necessary information required for recognition when facial expressions are presented dynamically. However, future work is necessary to examine the relationship between these scanning patterns and emotion recognition outcomes. 

For angry and neutral faces, infants exhibited a greater overall preference towards the eye compared to the mouth region, but for fearful and sad faces, there was not an overall difference in eye and mouth scanning. However, the time course analysis revealed that at the beginning of each trial, infants allocated more attention to the eye region compared to the mouth region across all dynamic expressions. These results are consistent with the literature on critical features of faces more generally, both in infancy and adulthood (i.e., preferential attention towards the eye region by 2 months of age, and a preference for open eyes compared to closed eyes; [21,22]). In adults, up to 70% of face fixations are directed towards the eyes, with the rest of their looks directed at the mouth and nose [14]. Eyes are a salient facial feature, and have been suggested to provide critical information that requires quick and efficient processing (e.g., direction of social gaze can be used to decipher important cues related to object location and may provide information regarding one’s mental state [48,49]), irrespective of the emotional expression portrayed [13]. These results suggest that sensitivity to the eyes as an important information source emerges early in development and is robust across dynamic expressions. Regarding fearful and sad faces, although the eye region is generally supported as the diagnostic region of these expressions for static faces, these expressions include greater mouth movement compared to angry and neutral faces. It is possible that when presented dynamically, infant’s attention is initially drawn to the critical, diagnostic region of the eyes, followed by a period of shifting to explore the mouth as it moves into the peak expression. Furthermore, given that neutral faces may be more ambiguous to infants, and they also have less experience with angry expressions [50], a possible interpretation of this finding may be that infants continue to allocate greater attention to the eye regions of these expressions in an effort to extract emotional information from these less expressive and less familiar expressions. This is in line with the finding that infants return to greater scanning of the eye region of neutral faces towards the end of the 3000 ms time window, after they returned from distributing their attention more equally between the eyes and mouth.

In addition, the time-course analysis further revealed that differential looking to the mouth only occurs after the eye region is initially attended. This finding is particularly relevant in the context of understanding visual exploration of happy expressions, as it provides a more refined picture than the mean dwell time analysis alone. The eye region of happy faces was attended to initially (similar to the other emotions) before the mouth region becomes the more prominent attentional target, which happens slightly later in the trial (around 1600 ms). Drawing on the saliency analysis, infants’ attention may be initially driven to the more salient eye region, but their attention is drawn to the mouth region later in the trial, despite this being a less visually salient area at the peak expression. Another important consideration is that greater looking to the mouth of happy faces emerged long after the dynamic expressions reached their peak, which occurred during the first 1000 ms of the trial. Given that increased looking to the mouth of happy faces occurred during the *second* 1000 ms of the trial, when the expression was held constant, it suggests that this increase is not simply driven by that area of the face being more mobile (i.e., as smiles move more than the mouths of other facial expressions). Furthermore, there is much greater looking to the mouth of happy expressions after 1000 ms compared to any other expression, despite the fact that other facial expressions show increased visual salience of the mouth region compared to happy faces (e.g., fear, sadness, and neutral). Despite the added value of analyzing visual salience in interpreting this finding, this analysis did not directly account for other salient properties of the stimuli (e.g., degree of novelty of the expressive face compared to the neutral expression, amount of movement during the shift from neutral to peak expression), which may have also contributed to infants’ looking patterns. 

Another finding that emerged in the current study was the main effect of emotion in the critical features analysis, such that infants exhibited greater attention to the happy face overall across both features. Although we did not have specific predictions about infants’ *overall* looking toward different emotions, increased attention towards this particular expression is supported by previous literature. In typical environments, infants receive increased exposure to happy expressions in early infancy [5,50]. Theories about the role of experience in the development of emotion recognition posit that greater exposure to a particular emotion category may enhance perceptual representations of that expression [51], which may subsequently lead to enhanced sensitivity to that expression (see [52,53] for examples in the context of anger and maltreatment). As such, infants’ greater allocation of attention towards happy expressions may be reflective of increased experience with this expression.

The stimuli used in this study limit the generalizability of the current results, despite being part of a validated stimulus set [42]. In particular, the current study presented infants with only female models. There may be differences in the ways that males and females express emotions [54,55], as well as infants’ ability to recognize emotions expressed by males and females [56,57]. As such, the current findings may only generalize to emotional expressions posed by females. Moreover, another important limitation relates to the mismatch between our diverse sample of participants and our selection of White models to express the emotions. Differences in infants’ processing of own- versus other-race faces is a well-studied phenomenon [58]. Experience with certain facial types can lead to important differences in subsequent face processing [56,59] (e.g., increased attention towards the internal facial features of own-race compared to other-race faces in 4- to 9-month-old infants [60] and it is likely that our diverse group of infants varied on their exposure to and familiarity with White faces. As a result, it is possible that our pattern of results regarding the ways in which infants scan dynamic expressions may be confounded by differences in the ways that White and non-White infants in the study scanned the faces, which were not controlled for in the current study. Furthermore, the ADFES stimulus set was validated by a group of White participants [42], which brings into question the validity of various properties of the expressions (e.g., valence, arousal) for non-White infants and may have further impacted our results. Future studies would benefit from including a more diverse stimulus set, so that infants are able to view faces that match the ethnicity of their primary caregiver.

Importantly, future work is required to examine whether infants’ online scanning patterns, as outlined in this paper, relate to other outcome variables, such as habituation, discrimination, and categorization of facial expressions of emotion. Broadening the current focus to include examination of abilities that are more closely tied to emotion recognition will allow for an even deeper understanding of the mechanisms driving these abilities.

## 5. Conclusions

In summary, the results from this study suggest that by 7 months of age, infants differentially allocate attention to facial features depending on the emotional expression. Infants allocated greater overall attention to the eyes compared to the mouth of angry and neutral faces, and they exhibited similar attention to the eyes and mouth of fearful and sad faces. When scanning happy faces, infants exhibited greater looking to the mouth compared to the eye region, which consisted of initial scanning of the eye region before attending to the mouth. The results of the current study contribute to our understanding of emotion processing by shedding light on the facial features that capture infants’ attention, and how this differs across emotions. When interpreted in tandem with other studies in the field that examine emotion recognition more directly, the results provided here may offer a tentative mechanism for understanding how infants learn to distinguish between different emotional expressions. 

## Figures and Tables

**Table 1:** Median saliency values for each feature of interest for the different emotional expressions.

	Anger	Fear	Happiness	Neutral	Sadness
Left eye	6	8	4	5	5
Right eye	7	11	8	7	8
Mouth	11	7	10	11	6

Note. Lower numbers indicate higher salience.

## Supplementary Materials

The following are available online at https://www.mdpi.com/2076-3425/10/9/585/s1. Figure S1: visual saliency maps for all expressions and all models.

## Appendix A

**Table A1:** Size (pixels) of interest areas.

Feature	Model 1	Model 2	Model 3	Model 4	Model 5
Right eye	10,976	11,760	11,160	10,622	12,100
Left Eye	10,976	11,760	11,160	10,622	12,100
Mouth	9380	8932	9246	9782	13,695
Face	72,909	75,703	77,829	74,623	101,509
Screen	415,582	415,582	415,582	415,582	415,582

Note. Areas of interest areas are consistent across all emotions for each model.

**Table A2:** Size of interest areas as a proportion of total screen size.

Feature	Model 1	Model 2	Model 3	Model 4	Model 5
Right eye	0.026	0.028	0.027	0.026	0.029
Left Eye	0.026	0.028	0.027	0.026	0.029
Mouth	0.023	0.021	0.022	0.024	0.033
Face	0.175	0.182	0.187	0.180	0.244
Screen	1	1	1	1	1

**Table A3:** Number of infants with ≥1 eye fixations per bin.

	Bin 1	Bin 2	Bin 3	Bin 4	Bin 5	Bin 6	Bin 7	Bin 8	Bin 9	Bin 10	Bin 11	Bin 12	Bin 13	Bin 14	Bin 15
Anger	27	37	37	42	43	43	47	43	43	42	39	34	31	29	37
Fear	25	36	35	40	39	41	38	29	30	30	30	36	37	36	38
Happy	31	38	32	37	41	45	34	21	15	17	18	18	18	18	26
Neutral	24	29	42	41	45	46	44	46	47	47	37	36	37	39	39
Sad	21	27	27	41	47	36	40	32	27	26	28	28	28	23	26

**Table A4:** Number of infants with ≥1 mouth fixations per bin.

	Bin 1	Bin 2	Bin 3	Bin 4	Bin 5	Bin 6	Bin 7	Bin 8	Bin 9	Bin 10	Bin 11	Bin 12	Bin 13	Bin 14	Bin 15
Anger	0	0	2	2	3	5	4	3	3	9	13	10	10	12	9
Fear	0	0	3	4	3	4	4	9	12	15	15	16	15	11	10
Happy	0	0	1	7	7	7	7	11	21	28	31	33	29	28	22
Neutral	0	0	2	6	4	4	3	3	6	6	7	8	7	5	6
Sad	1	0	1	4	3	5	6	8	13	18	17	14	16	14	13

## References

[1] Nelson C.A. (1987). The Recognition of Facial Expressions in the First Two Years of Life: Mechanisms of Development. Child Dev..

[2] Wagner J.B., Luyster R.J., Yim J.Y., Tager-Flusberg H., Nelson C.A. (2013). The role of early visual attention in social development. Int. J. Behav. Dev..

[3] Legerstee M. (2009). The role of dyadic communication in social cognitive development. Adv. Child Dev. Behav..

[4] Nelson C.A., De Haan M. (1996). Neural correlates of infants’ visual responsiveness to facial expressions of emotion. Dev. Psychobiol..

[5] Farroni T., Menon E., Rigato S., Johnson M.H. (2007). The perception of facial expressions in newborns. Eur. J. Dev. Psychol..

[6] Bornstein M.H., Arterberry M.E. (2003). Recognition, discrimination and categorization of smiling by 5-month-old infants. Dev. Sci..

[7] Walker-Andrews A.S. (1988). Infants’ perception of the affordances of expressive behaviors. Advances in Infancy Research.

[8] Walker-Andrews A.S. (1986). Intermodal perception of expressive behaviors: Relation of eye and voice?. Dev. Psychol..

[9] Walker A.S. (1982). Intermodal perception of expressive behaviors by human infants. J. Exp. Child Psychol..

[10] Klinnert M.D. (1984). The regulation of infant behavior by maternal facial expression. Infant Behav. Dev..

[11] Amso D., Fitzgerald M., Davidow J., Gilhooly T., Tottenham N. (2010). Visual Exploration Strategies and the Development of Infants’ Facial Emotion Discrimination. Front. Psychol..

[12] Xiao N.G., Quinn P.C., Liu S., Ge L., Pascalis O., Lee K. (2015). Eye tracking reveals a crucial role for facial motion in recognition of faces by infants. Dev. Psychol..

[13] Scheller E., Buchel C., Gamer M. (2012). Diagnostic Features of Emotional Expressions Are Processed Preferentially. PLoS ONE.

[14] Walker-Smith G.J., Gale A.G., Findlay J.M. (1977). Eye Movement Strategies Involved in Face Perception. Perception.

[15] Adolphs R., Gosselin F., Buchanan T.W., Tranel D., Schyns P., Damasio A.R. (2005). A mechanism for impaired fear recognition after amygdala damage. Nat..

[16] Boucher J.D., Ekman P. (1975). Facial Areas and Emotional Information. J. Commun..

[17] Eisenbarth H., Alpers G.W. (2011). Happy mouth and sad eyes: Scanning emotional facial expressions. Emotion.

[18] Hanawalt N.G. (1944). The RÔle of the Upper and the Lower Parts of the Face as a Basis for Judging Facial Expressions: II. In Posed Expressions and “Candid-Camera” Pictures. J. Gen. Psychol..

[19] Schurgin M.W., Nelson J., Iida S., Ohira H., Chiao J.Y., Franconeri S.L. (2014). Eye movements during emotion recognition in faces. J. Vis..

[20] Smith M.L., Cottrell G.W., Gosselin F., Schyns P.G. (2005). Transmitting and Decoding Facial Expressions. Psychol. Sci..

[21] Batki A., Baron-Cohen S., Wheelwright S., Connellan J., Ahluwalia J. (2000). Is there an innate gaze module? Evidence from human neonates. Infant Behav. Dev..

[22] Maurer D., Field T., Fox N. (1985). Infants’ perception of facedness. Social Perception in Infants.

[23] Oakes L.M., Ellis A.E. (2011). An Eye-Tracking Investigation of Developmental Changes in Infants’ Exploration of Upright and Inverted Human Faces. Infancy.

[24] Miguel H.O., McCormick S.A., Westerlund A., Nelson C.A. (2019). Rapid face processing for positive and negative emotions in 5-, 7-, and 12-month-old infants: An exploratory study. Br. J. Dev. Psychol..

[25] Gredebäck G., Eriksson M., Schmitow C., Laeng B., Stenberg G. (2011). Individual Differences in Face Processing: Infants’ Scanning Patterns and Pupil Dilations are Influenced by the Distribution of Parental Leave. Infancy.

[26] Peltola M.J., Leppänen J.M., Vogel-Farley V.K., Hietanen J.K., Nelson C.A. (2009). Fearful faces but not fearful eyes alone delay attention disengagement in 7-month-old infants. Emotion.

[27] Hunnius S., De Wit T.C.J., Vrins S., Von Hofsten C. (2011). Facing threat: Infants’ and adults’ visual scanning of faces with neutral, happy, sad, angry, and fearful emotional expressions. Cogn. Emot..

[28] Jessen S., Altvater-Mackensen N., Grossmann T. (2016). Pupillary responses reveal infants’ discrimination of facial emotions independent of conscious perception. Cognition.

[29] Quinn P.C., Anzures G., Izard C.E., Lee K., Pascalis O., Slater A.M., Tanaka J.W. (2011). Looking Across Domains to Understand Infant Representation of Emotion. Emot. Rev..

[30] Heck A., Hock A., White H., Jubran R., Bhatt R.S. (2016). The development of attention to dynamic facial emotions. J. Exp. Child Psychol..

[31] Geangu E., Hauf P., Bhardwaj R., Bentz W. (2011). Infant Pupil Diameter Changes in Response to Others’ Positive and Negative Emotions. PLoS ONE.

[32] Kim H.I., Johnson S.P. (2013). Do young infants prefer an infant-directed face or a happy face?. Int. J. Behav. Dev..

[33] Caron R.F., Caron A.J., Myers R.S. (1985). Do Infants See Emotional Expressions in Static Faces?. Child Dev..

[34] Soken N.H., Pick A.D. (1992). Intermodal Perception of Happy and Angry Expressive Behaviors by Seven-Month-Old Infants. Child Dev..

[35] Bassili J.N. (1978). Facial motion in the perception of faces and of emotional expression. J. Exp. Psychol. Hum. Percept. Perform..

[36] Wilcox B.M., Clayton F.L. (1968). Infant visual fixation on motion pictures of the human face. J. Exp. Child Psychol..

[37] Otsuka Y., Konishi Y., Kanazawa S., Yamaguchi M.K., Abdi H., O’Toole A.J. (2009). Recognition of Moving and Static Faces by Young Infants. Child Dev..

[38] Soussignan R., Dollion N., Schaal B., Durand K., Reissland N., Baudouin J.-Y. (2017). Mimicking emotions: How 3–12-month-old infants use the facial expressions and eyes of a model. Cogn. Emot..

[39] Kestenbaum R., Nelson C.A. (1990). The recognition and categorization of upright and inverted emotional expressions by 7-month-old infants. Infant Behav. Dev..

[40] Ludemann P.M., Nelson C.A. (1988). Categorical representation of facial expressions by 7-month-old infants. Dev. Psychol..

[41] Faul F., Erdfelder E., Lang A.-G., Buchner A. (2007). G*Power 3: A flexible statistical power analysis program for the social, behavioral, and biomedical sciences. Behav. Res. Methods.

[42] Van Der Schalk J., Hawk S.T., Fischer A.H., Doosje B. (2011). Moving faces, looking places: Validation of the Amsterdam Dynamic Facial Expression Set (ADFES). Emot..

[43] Benjamini Y., Hochberg Y. (1995). Controlling the False Discovery Rate: A Practical and Powerful Approach to Multiple Testing. J. R. Stat. Soc. Ser. B.

[44] Hood B.M., Atkinson J. (1993). Disengaging visual attention in the infant and adult. Infant Behav. Dev..

[45] Walther D., Koch C. (2006). Modeling attention to salient proto-objects. Neural Netw..

[46] Ichikawa H., Kanazawa S., Yamaguchi M.K. (2014). Infants recognize the subtle happiness expression. Perception.

[47] Ichikawa H., Yamaguchi M.K. (2013). Infants’ recognition of subtle anger facial expression. Jpn. Psychol. Res..

[48] Emery N.J. (2000). The eyes have it: The neuroethology, function and evolution of social gaze. Neurosci. Biobehav. Rev..

[49] Langton S.R.H., Watt R.J., Bruce V. (2000). Do the eyes have it? Cues to the direction of social attention. Trends Cogn. Sci..

[50] Malatesta C.Z., Haviland J.M. (1982). Learning Display Rules: The Socialization of Emotion Expression in Infancy. Child Dev..

[51] Leppänen J.M., Nelson C.A. (2008). Tuning the developing brain to social signals of emotions. Nat. Rev. Neurosci..

[52] Pollak S.D., Cicchetti D., Hornung K., Reed A. (2000). Recognizing emotion in faces: Developmental effects of child abuse and neglect. Dev. Psychol..

[53] Pollak S.D., Klorman R., Thatcher J.E., Cicchetti D. (2001). P3b reflects maltreated children’s reactions to facial displays of emotion. Psychophysiol..

[54] Hall J.A. (1984). Nonverbal Sex Differences: Accuracy of Communication and Expressive Style.

[55] Lafrance M., Hecht M.A., Paluck E.L. (2003). The contingent smile: A meta-analysis of sex differences in smiling. Psychol. Bull..

[56] Bayet L., Quinn P.C., Tanaka J.W., Lee K., Gentaz E., Pascalis O. (2015). Face Gender Influences the Looking Preference for Smiling Expressions in 3.5-Month-Old Human Infants. PLoS ONE.

[57] Montague D.R.F., Walker-Andrews A.S. (2002). Mothers, Fathers, and Infants: The Role of Person Familiarity and Parental Involvement in Infants’ Perception of Emotion Expressions. Child Dev..

[58] Kelly D.J., Quinn P.C., Slater A.M., Lee K., Gibson A., Smith M., Ge L., Pascalis O. (2005). Three-month-olds, but not newborns, prefer own-race faces. Dev. Sci..

[59] Quinn P.C., Yahr J., Kuhn A., Slater A.M., Pascalis O. (2002). Representation of the Gender of Human Faces by Infants: A Preference for Female. Perception.

[60] Liu S., Quinn P.C., Wheeler A., Xiao N., Ge L., Lee K. (2011). Similarity and difference in the processing of same- and other-race faces as revealed by eye tracking in 4- to 9-month-olds. J. Exp. Child Psychol..

