# Supplementary material for: Dynamic Advances in Emotion Processing: Differential Attention towards the Critical Features of Dynamic Emotional Expressions in 7-Month-Old Infants
Source: Brain Sci. 2020 Aug 24;10(9):585. doi: 10.3390/brainsci10090585 (PMC7564740; doi:10.3390/brainsci10090585)

Supplementary Figure S1. Heat maps representing visual saliency derived from the Matlab Saliency Toolbox [44] overlaid on peak facial expressions for the five identities used in the current study. Hot colors (e.g., red, yellow) represent higher saliency locations and cool colors (e.g., blue) represent lower saliency locations.

A) Anger

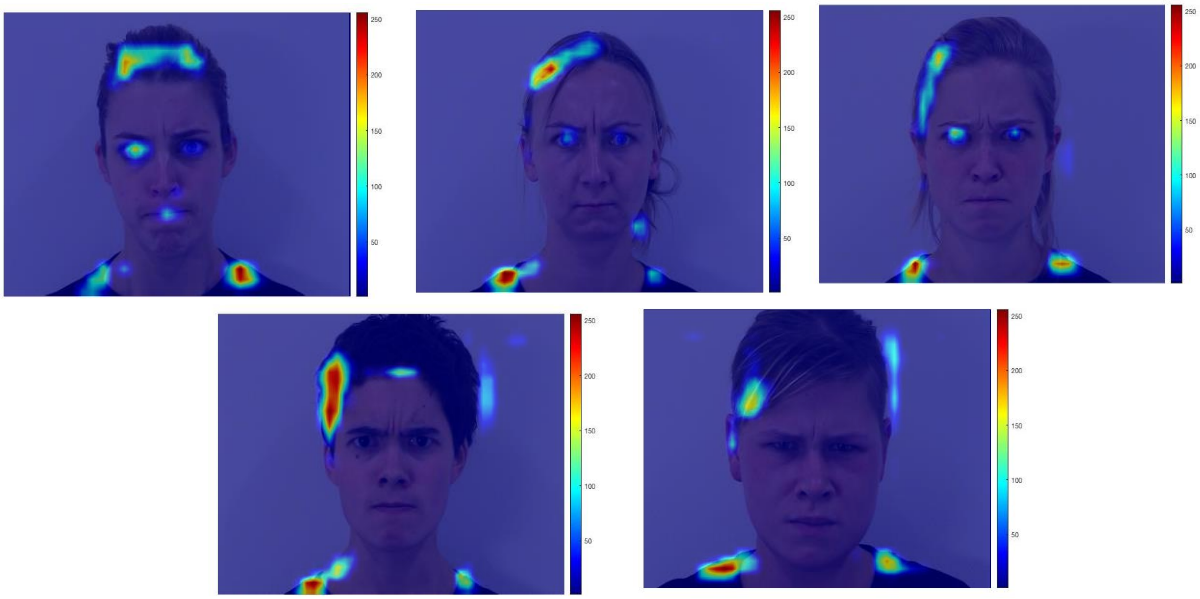

B) Fear

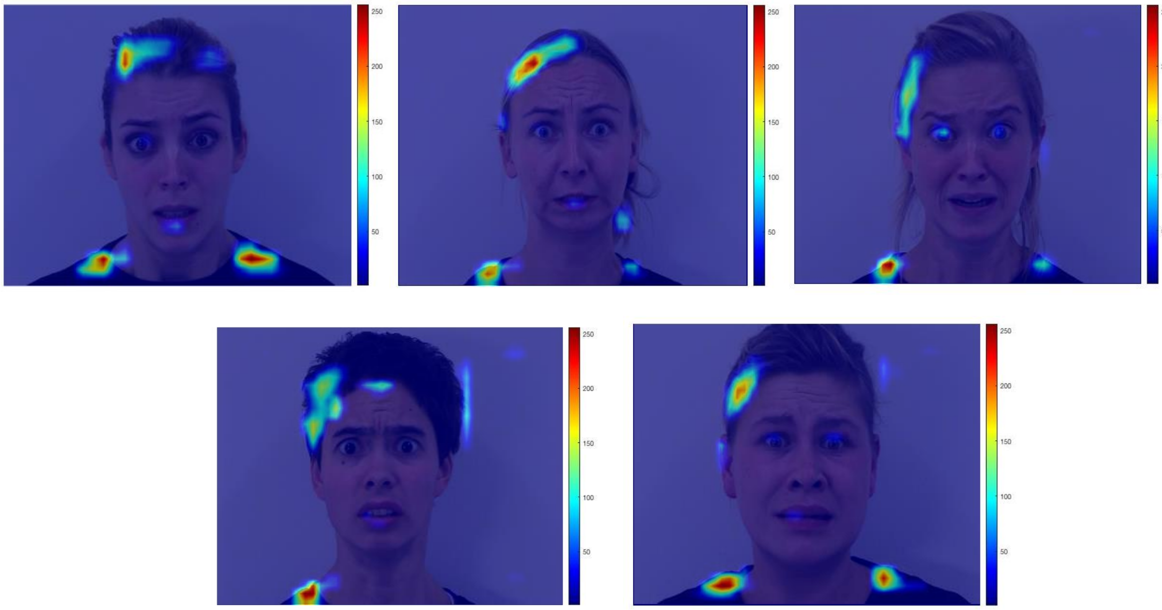

C) Happiness

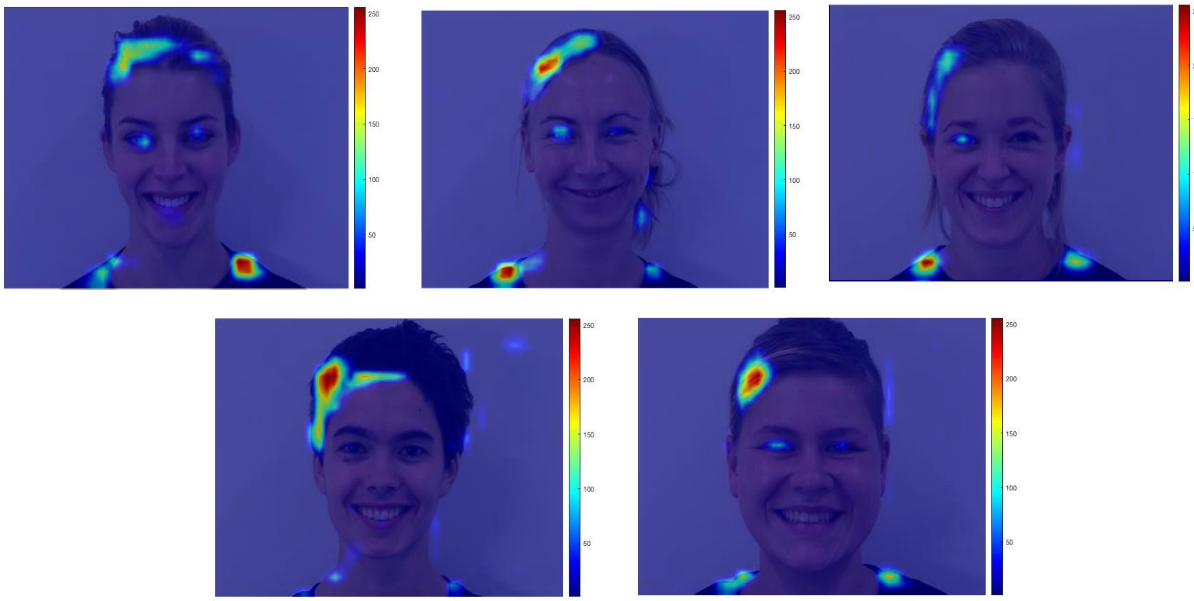

D) Neutral

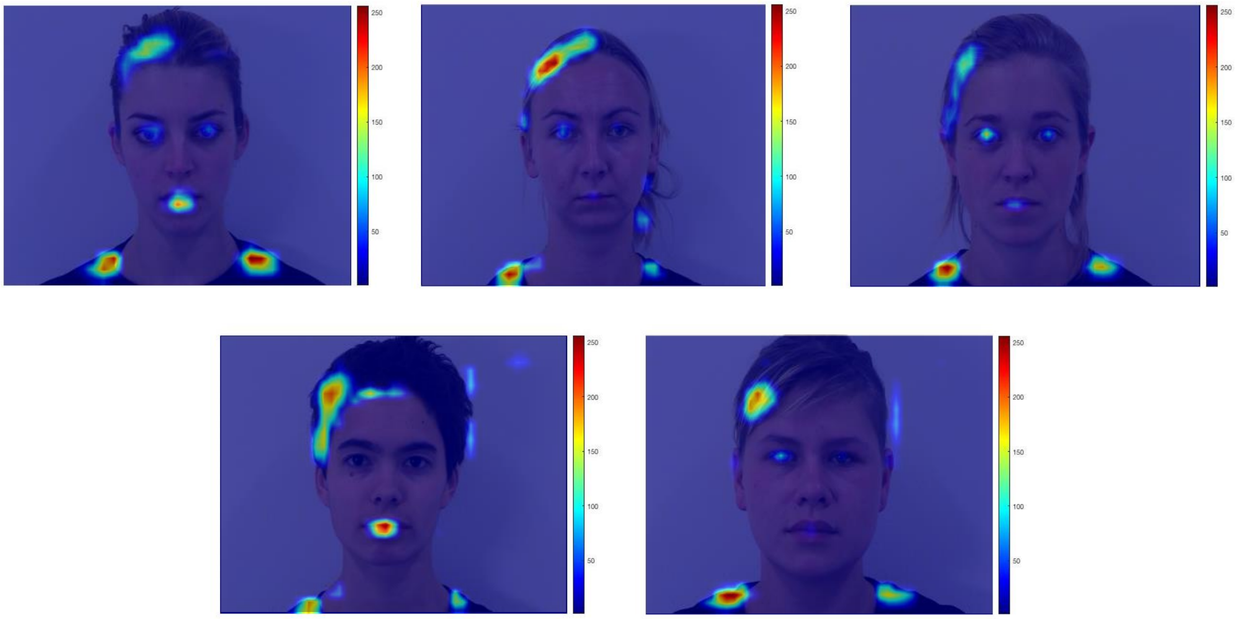

E) Sadness

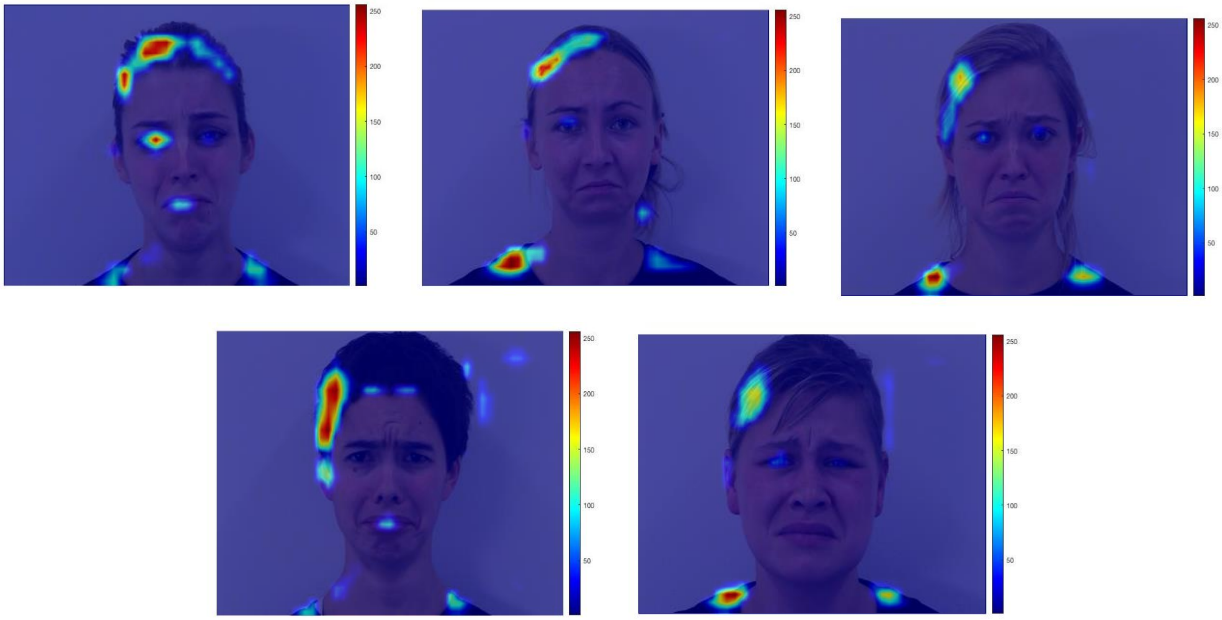

Supplement: Supplementary file 1 [file brainsci-10-00585-s001.pdf]
